# Stable C and N isotope natural abundances of intraradical hyphae of arbuscular mycorrhizal fungi

**DOI:** 10.1007/s00572-020-00981-9

**Published:** 2020-08-25

**Authors:** Saskia Klink, Philipp Giesemann, Timo Hubmann, Johanna Pausch

**Affiliations:** 1 Department of Agroecology, Bayreuth Center of Ecology and Environmental Research (BayCEER), University of Bayreuth, 95440 Bayreuth, Germany; 2 Laboratory of Isotope Biogeochemistry, Bayreuth Center of Ecology and Environmental Research (BayCEER), University of Bayreuth, 95440 Bayreuth, Germany

**Keywords:** Mycorrhiza, Hyphae, δ^13^C, δ^15^N, Nitrogen acquisition, Plant carbon

## Abstract

Data for stable C and N isotope natural abundances of arbuscular mycorrhizal (AM) fungi are currently sparse, as fungal material is difficult to access for analysis. So far, isotope analyses have been limited to lipid compounds associated with fungal membranes or storage structures (biomarkers), fungal spores and soil hyphae. However, it remains unclear whether any of these components are an ideal substitute for intraradical AM hyphae as the functional nutrient trading organ. Thus, we isolated intraradical hyphae of the AM fungus *Rhizophagus irregularis* from roots of the grass *Festuca ovina* and the legume *Medicago sativa* via an enzymatic and a mechanical approach. In addition, extraradical hyphae were isolated from a sand-soil mix associated with each plant. All three approaches revealed comparable isotope signatures of *R. irregularis* hyphae. The hyphae were ^13^C- and ^15^N-enriched relative to leaves and roots irrespective of the plant partner, while they were enriched only in ^15^N compared with soil. The ^13^C enrichment of AM hyphae implies a plant carbohydrate source, whereby the enrichment was likely reduced by an additional plant lipid source. The ^15^N enrichment indicates the potential of AM fungi to gain nitrogen from an organic source. Our isotope signatures of the investigated AM fungus support recent findings for mycoheterotrophic plants which are suggested to mirror the associated AM fungi isotope composition. Stable isotope natural abundances of intraradical AM hyphae as the functional trading organ for bi-directional carbon-for-mineral nutrient exchanges complement data on spores and membrane biomarkers.

**Electronic supplementary material:**

The online version of this article (10.1007/s00572-020-00981-9) contains supplementary material, which is available to authorized users.

## Introduction

Natural abundances of stable isotopes are used to trace carbon and both inorganic and organic nitrogen fluxes within plants, fungi and their associations (e.g. Gleixner et al. 1993; Gebauer and Dietrich 1993; Courty et al. 2015; Chen et al. 2019; Giesemann et al. 2020; Suetsugu et al. 2020). While profound isotopic data exist for ectomycorrhizal and saprotrophic fungi, information on arbuscular mycorrhizal (AM) fungi remain sparse due to the limited accessibility of sporocarps and the fragile nature of hyphae.

Nevertheless, a few data on isotope natural abundances are available from spores, soil hyphae and biomarkers such as phospholipid fatty acids (PLFAs) or neutral lipid fatty acids (NLFAs) (Allen and Allen 1990; Nakano et al. 1999; Courty et al. 2011, 2015; Walder et al. 2012, 2013; Suetsugu et al. 2020). Still, it is unclear whether spores and PLFA/NLFA biomarkers mirror intraradical hyphae, the functional trading organ. For instance, Courty et al. (2015) assumed the carbon (^13^C) isotopic signature of spores might be variable because of variable lipid storage and therefore that spores may not represent an ideal substitute for AM hyphal tissue.

Mycorrhizal fungi are supplied with ^13^C-enriched carbon from their plant partner (Gleixner et al. 1993; Gebauer et al. 2016). Thus, we expect a ^13^C enrichment for AM hyphae relative to plant bulk material. Such ^13^C enrichment for AM hyphae was shown by Walder et al. (2012), although their work was subject to the methodological challenge of separating soil hyphae from soil contaminants (Hodge and Fitter 2010; Walder et al. 2012). We report a dual stable ^13^C and nitrogen (^15^N) isotope natural abundance approach to separate both soil hyphae and intraradical hyphae from soil or plant contaminants.

Knowledge of AM intraradical hyphal ^15^N isotope natural abundances will enable the deciphering of nitrogen sources for fungal nutrition. Previously, ^15^N isotopic signatures were used to evaluate organic vs inorganic nitrogen nutrient sources for plants and fungi (Gebauer and Dietrich 1993; Schulze et al. 1994; Michelsen et al. 1996, 1998). Arbuscular mycorrhizal fungi may acquire nitrogen from isotopically inconspicuous inorganic ammonium and nitrate, like plant roots (Field and Pressel 2018), or a mixture of inorganic and ^15^N-enriched organic nitrogen nutrients (Gebauer and Dietrich 1993) released by saprotrophic organisms (Leigh et al. 2009; Hodge and Fitter 2010). AM fungi are commonly considered to have limited saprotrophic capabilities (Nakano et al. 1999; Smith and Read 2008; Tisserant et al. 2013), although nitrogen acquisition from organic patches by AM fungi has been shown (Hodge et al. 2001; Leigh et al. 2009). These different nitrogen sources likely influence the ^15^N pattern of AM hyphae. Thus, ^15^N-signatures will provide further information on the relative importance of inorganic versus organic nitrogen as nutrient sources for AM fungi.

To address these uncertainties, in this study we present for the first time dual stable ^13^C and ^15^N isotope natural abundances of intraradical AM fungal hyphae. Intraradical hyphae were isolated using two distinct methods, a mechanical and an enzymatic approach. The results were then compared with the stable isotope composition of AM soil hyphae collected by sieving. In addition, the fungal signatures were compared with the associated plant partner tissues’ signatures to assess relative isotopic enrichments. We hypothesize that intraradical hyphae and soil hyphae are ^13^C-enriched relative to plant material while the acquisition of ^15^N-inconspicuous ammonium and nitrate will result in equal ^15^N isotope abundances for plant material and fungal hyphae. Furthermore, we hypothesize the dual isotope approach will improve the separation of plant, fungal and soil compounds. The feasibility of the isolation approach and the quantifiability of intraradical hyphae as the functional nutrient trading organ versus existing approaches using spores or PLFAs/NLFAs as specific fungal components are discussed.

## Materials and methods

### Experimental set-up

**Fig. 1:**
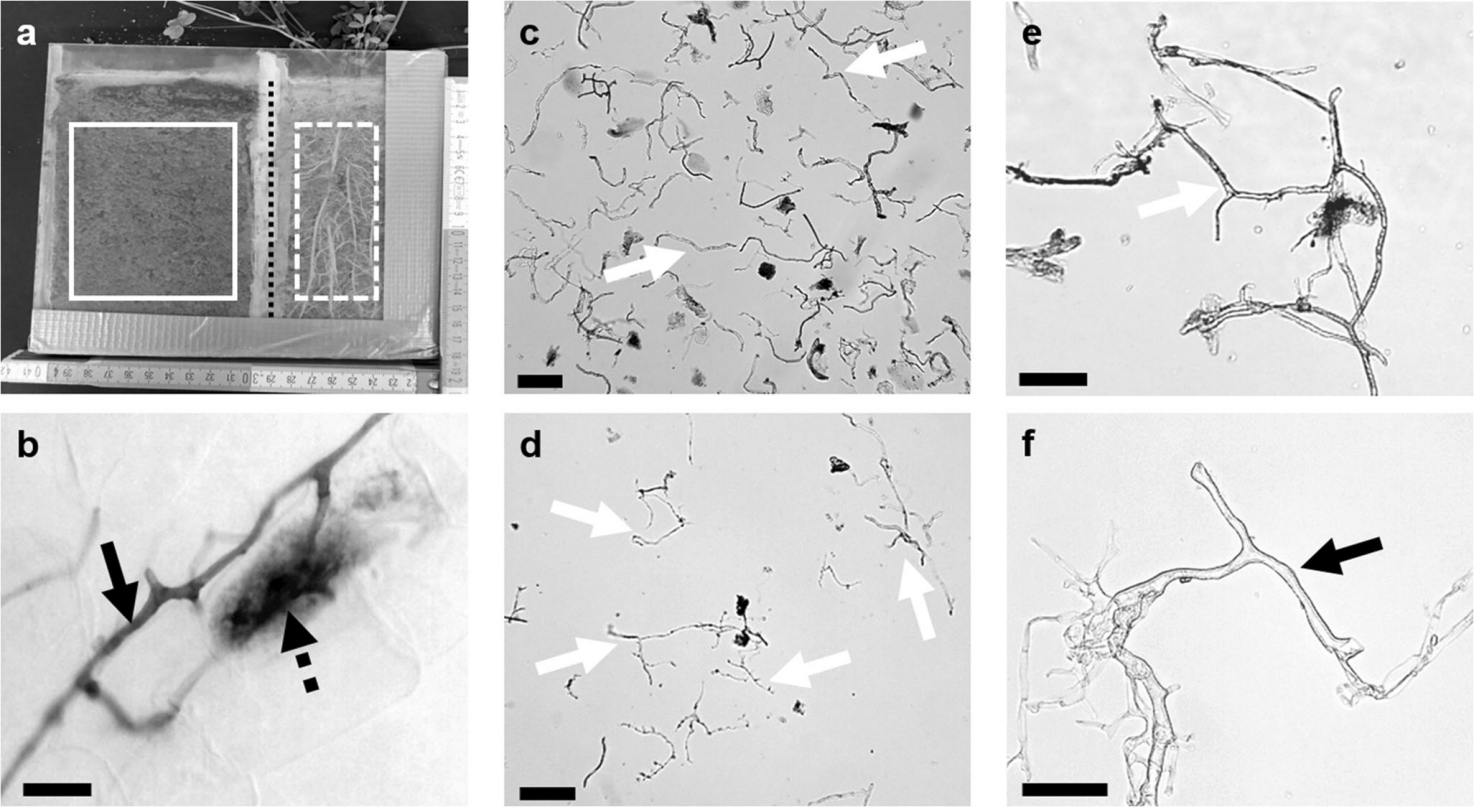
Collage of split rhizoboxes and AM hyphae isolation procedure. (**a**) Side view of *Medicago sativa* planted in a split rhizobox. The white box illustrates the hyphae-only compartment (HC), the white dashed box the plant root compartment (PC), both separated by a 30 μm pore size mesh (dashed black line). (**b**) Hyphae of AMF (black arrow) and arbuscule (dashed arrow) within root cells. (**c**) Soil hyphae (white arrows) and organic material after suspending in sodium-hexametaphosphate, (**d**) soil hyphae (white arrows) after cleaning and sieving steps. (**e**) AMF hyphae (white arrow) from soil, (**f**) AMF hyphae (black arrow) from root extraction. Scale **b**, **f** 10 μm; **c**–**e** 100 μm

The grass *Festuca ovina* L. and the legume *Medicago sativa* L. were planted separately in split-rhizoboxes (six rhizoboxes per species; 20 × 20 × 3 cm) with removable front covers (Fig. 1a). The boxes were separated into a plant roots compartment (PC) and a hyphae-only compartment (HC) via a 30 μm mesh (Sefar Nitex PA, 03-30/18, Heiden, Switzerland) and were filled with a sterilized (121 °C, 200 kPa; Systec DE45; Systec GmbH, Linden, Germany) soil-sand-mixture (1:1, v/v; sieved to 2 mm). The soil was taken from a grassland site (upper 10 cm) at the Landwirtschaftlichen Lehranstalten Bayreuth (49° 55′ 42.618″ N, 11° 33′ 2.8656″ E) and is classified as a sandy clay loam with 0.1% nitrogen, 1.0% carbon and a pH of 5.3.

Prior to filling the boxes, the initially steam-sterilized soil-sand-substrate was mixed with spores of *Rhizophagus irregularis* (AMM 6080001, BioFA AG, Münsingen, Germany). Spores were separated from the culture substrate by mixing approx. 1 g substrate with 50 mL sterile water and decanting the supernatant on top of a combination of stacked sieves of 250 μm, 90 μm and 20 μm before spores settled down (adapted from Cranenbrouck et al. (2005)). The fractions on the 90 μm and 20 μm sieves were transferred to centrifuge tubes and centrifuged (3 min at 2000*g*, Centrifuge 5810R, Eppendorf AG, Hamburg, Germany) before spores were collected from the bottom of the tube while the supernatant with substrate remains was discarded. Using a vacuum filtration, spores were surface-sterilized (4% hydrogen peroxide for 10 min) and rinsed with sterile deionized water three times. Spores obtained from 1 g culture substrate (approx. 400 spores) were homogeneously added to 1230 g sand-soil mix filling both compartments of the rhizobox. The rhizobox was covered with aluminium foil. The water holding capacity of the sand-soil mix was maintained at 60% throughout the duration of the growing period (78 days).

Seeds (Jelitto Staudensamen GmbH, Schwarmstedt, Germany) of *F. ovina* and *M. sativa* were surface-sterilized (5 min in 6% hydrogen peroxide) and germinated on sterile moist filter paper. Two plants per box, either the grass or the legume, were planted into the PC. Plants were grown in a climate-control chamber (Adaptis A100, Conviron, China) at 26 °C/22 °C day/night with a light period of 14 h and mean photosynthetically active radiation (PAR) flux of 200 μmol photons m^−2^ s^−1^. After the growth period, the rhizoboxes were destructively harvested. The sand-soil mix from the HC was gently sieved to 2-mm to remove coarser organic particles and stored in a refrigerator (4 °C). Isolation of soil hyphae was done immediately after harvest. The PC was separated into above- and belowground biomass which was washed with deionized water and cleaned with tweezers. Samples of leaves and subsamples of roots and soil (HC) were dried to constant weight (48 h at 60 °C) and ground (ballmill MM200, Retsch GmbH, Haan, Germany) to fine powder for isotope analyses.

### Isolation of intraradical AM hyphae

Intraradical hyphae of *R. irregularis* (PC) were isolated via an enzymatic approach modified from Saito (1995) and via a novel mechanical approach which aimed to avoid chemical-induced isotopic fractionation. Microscopic observation (Motic BA210; Fig. 1b–f) of aseptate, hyaline hyphae, seldom accompanied by arbuscules, vesicles and spores between the procedures was always a key step to evaluate the success of hyphal isolation.

According to our alteration from Saito (1995), roots cut into 5 mm segments and washed with deionized water were sonicated (35 kHz; Bandelin, Sonorex RK100H, Berlin, Germany) to remove soil residues and external hyphae. Constituents of the enzyme solution were 20 g L^−1^ Cellulase ‘Onozuka’ RS (SERVA Electrophoresis GmbH, Heidelberg, Germany), 1 g L^−1^ Pectolyase ‘Y23’ (Sigma-Aldrich, Darmstadt, Germany) in 0.01 M MES-KOH pH 5.5 buffer (Carl Roth, Karlsruhe, Germany). The penetration rate of the enzyme solution was increased by sonication (10 min at 20 °C) instead of using an aspirator. If not mentioned otherwise, all steps were performed at 4 °C. For the mechanical isolation, 5 mm root segments were washed with deionized water; root cell layers opened with scalpels by slicing sagittally while holding with tweezers, followed by sonication (30 min at 20 °C) in deionized water to release hyphae from the sliced roots into the water column. To compare intraradical and extraradical AM hyphae, soil hyphae (HC) were isolated according to Brundrett et al. (1994), using the whole sample (5 g soil) instead of an aliquot to increase the recovery of hyphae in a suitable amount for isotopic measurement.

Cleaning steps were identical for all samples of intraradical and soil hyphae. A combination of stacked 500 μm, 250 μm, 90 μm, 63 μm, and 20 μm sieves (Retsch test sieve, stainless steel, DIN/ISO 3310-1, Germany) separated hyphae from coarse roots, plant residues and soil particles, whereby sodium-hexametaphosphate and enzyme solution remains were washed out. To ensure a high recovery of hyphae, the sieve surface was gently sprayed and rinsed with deionized water several times. Here, most hyphae were recovered from the 20 μm sieve. It is to be noted that when isolating hyphae of different morphologies, e.g. coils or pelotons, these large structures may necessitate a greater mesh size. Cleaned hyphae (Fig. 1d) were washed from the sieve into Eppendorf tubes and centrifuged (5 s at 1306×*g*, Eppendorf Centrifuge 5415 C, Eppendorf AG, Hamburg, Germany). Microscopic observation showed that both the supernatant and the pellet contained hyphae. Because the supernatant comprised clean hyphae, while the pellet was a mixture of hyphae and organic residue, hyphae were collected from the supernatant. This collection of hyphae had to be done quickly to recover hyphae before they settled to the bottom of the tube with the soil residues. The pellet was resuspended, and the centrifugation step repeated 4–5 times until no appreciable number of hyphae were microscopically observable either in the supernatant or in the pellet. A hyphal pellet then was produced by centrifugation (20 min at 3220×*g*, Centrifuge 5810R, Eppendorf AG, Hamburg, Germany) and dried at 60 °C for 72 h. Storage was in desiccators with silica gel until stable isotope analysis.

As an additional test for chemical-induced isotopic fractionation on fungal tissue with a greater sample mass than AMF hyphae, sporocarps of *Agaricus bisporus* (J. E. Lange) Imbach treated with either the enzyme or sodium-hexametaphosphate solution were compared with non-treated controls.

### Stable isotope analysis

Hyphal material (soil hyphae *n* = 8, intraradical hyphae: mechanic *n* = 8, enzymatic *n* = 4; *n* = 20 per plant species) was suspended in 200 μL deionized water, transferred into tin capsules (4 × 6 mm) and dried for 24 h at 60 °C. This procedure was repeated until the complete hyphal suspension was dried into the tin capsules. Due to the low weight of hyphae, the ‘N blank effect’, occurring when the O_2_ pulse is supplied in the elemental analyser isotope ratio mass spectrometer (EA-IRMS), can lead to increasingly inaccurate ^15^N values, while ^13^C is not affected (Crotty et al. 2013). Therefore, a subset of eight hyphal samples (soil hyphae *n* = 4; intraradical hyphae: mechanic *n* = 4; *n* = 2 per species) was analysed with a Micro Elemental Analyser Isotope Ratio Mass Spectrometer (μEA-IRMS), specialized for samples with a low weight and sample size. The results of these measurements show the combination of δ^15^N and δ^13^C values of AMF hyphae and corroborate the ^13^C data of soil hyphae and intraradical hyphae (both mechanic and enzymatic isolation) gained from the EA-IRMS measurement. Hyphal samples with unreliable signal intensity for δ^15^N were omitted for this study, resulting in a total of twelve hyphal samples for ^15^N (*n* = 3 for soil hyphae, *n* = 3 for mechanically isolated intraradical hyphae per plant species).

Plant leaves (*n* = 12 per plant species), roots (*n* = 6 per plant species), soil (HC, *n* = 6 per plant species), fruiting bodies of *Agaricus bisporus* (*n* = 24) and hyphae were analysed for stable isotope natural abundance of δ^13^C, δ^15^N and C- and N- concentrations at the BayCEER Laboratory of Isotope-Biogeochemistry (EA-IRMS; University of Bayreuth, Germany) and the Centre for Stable Isotope Research and Analysis (μEA-IRMS; Georg-August-University, Göttingen, Germany). The isotope abundances are expressed as δ-notation relative to Vienna-PDBelemite (^13^C standard) or air (^15^N standard): δ^13^C or δ^15^N = (*R*_sample_/*R*_standard_ − 1) × 1000 (‰), whereby *R* is the ratio of the heavy to the respective light isotope.

### Statistics

Software RStudio 1.2.5019 (RStudio Team 2019) was used for statistical analysis, and graphics were created with RStudio or SigmaPlot 11.0 (Systat Software, San Jose, USA). Shaprio-Wilk’s test for all data supported a non-parametric test procedure. One-tailed Kruskal-Wallis (*H*) tests followed by pairwise Wilcoxon post hoc test procedures (*Z*) were applied for differences across hyphal, plant and soil samples as well as across the hyphae isolation methods. *P* values were adjusted with a Holm-Bonferroni correction. Mann-Whitney *U* tests were applied to evaluate chemical-induced differences between treated samples and controls. The level of significance was *α* = 0.05.

## Results and discussion

### Isotopic patterns of AM hyphae relative to associated plants and soil

**Fig. 2:**
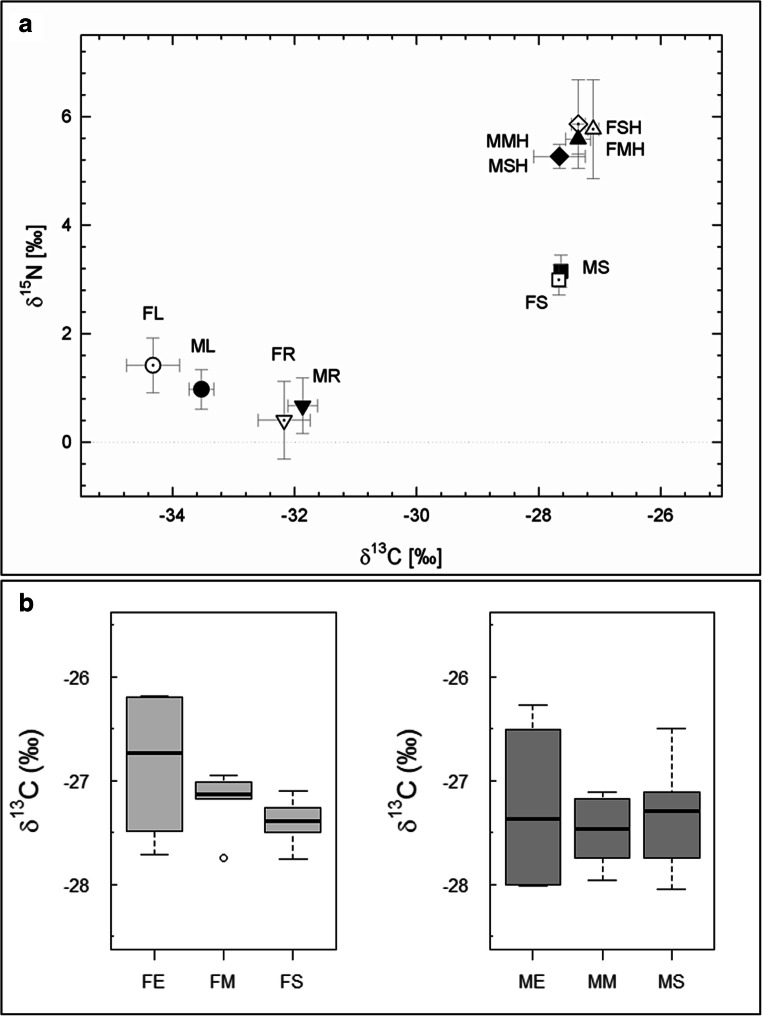
**a** Scatter plot of δ^13^C and δ^15^N stable isotope natural abundances of leaves (circles), roots (downwards triangles), soil (squares), soil hyphae (diamonds) and mechanically isolated intraradical hyphae (upwards triangles) from the grass *Festuca ovina* (white symbols) and the legume *Medicago sativa* (black symbols) microcosms. FL *Festuca* leaves, ML *Medicago* leaves, FR *Festuca* roots, MR *Medicago* roots, FS *Festuca* soil, MS *Medicago* soil, FSH *Festuca* soil hyphae, FMH *Festuca* mechanically isolated hyphae, MSH *Medicago* soil hyphae, MMH *Medicago* mechanically isolated hyphae. Error bars indicate standard deviation (SD). **b** Whisker boxplot of δ^13^C values of AM fungal hyphae from the grass (left) and the legume (right), respectively, shown for the different isolation methods (soil hyphae; intraradical hyphae: mechanic vs enzymatic). FS *Festuca* soil hyphae, MS *Medicago* soil hyphae, FM *Festuca* mechanically isolated hyphae, MM *Medicago* mechanically isolated hyphae, FE *Festuca* enzymatically isolated hyphae, ME *Medicago* enzymatically isolated hyphae. The black line in the centre of the box indicates the median, box margins represent the 25th and 75th percentiles. The length of the box is the inter quartile range (IQR), whiskers indicate the minimum (25th percentile − 1.5 × IQR) and the maximum (75th percentile + 1.5 × IQR). Outliers are shown as circles

Our data show for the first time, to our knowledge, stable δ^13^C and δ^15^N isotope natural abundances of intraradical AM hyphae (Fig. 2a). The isolated AM fungal material was *c*. 6.5‰ enriched relative to leaves and *c*. 4.6‰ relative to roots in ^13^C and *c*. 4.2‰ enriched relative to leaves and *c*. 4.9‰ relative to roots in ^15^N (Fig. 2a). The ^13^C signatures of soil and AM fungal material were clustered together yet showed distinct ^15^N enrichment with 2.4‰ higher ^15^N enrichment of the AM hyphae compared with the soil. No significant differences in hyphal δ^13^C were discovered among the three isolation approaches either for hyphae from the grass (*H* = 2.479, df = 2, *P* = 0.29) or from the legume (*H* = 0.106, df = 2, *P* = 0.95; Fig. 2b). The same holds true for δ^15^N comparing the two groups of soil hyphae isolated by sieving and the mechanically isolated intraradical hyphae (grass *U*(3,3) = 5, *P* = 1.000, legume *U*(3,3) = 2, *P* = 0.383; Fig. 2a). No influence of involved extracting agents was detected (Table S2). Hyphae samples were significantly enriched in δ^13^C and δ^15^N relative to leaves, roots and soil of *Festuca* (δ^13^C: *H* = 24.296, df = 3, *P* < 0.001; δ^15^N: *H* = 25.211, df = 3, *P* < 0.001) and of *Medicago* (δ^13^C: *H* = 22.734, df = 3, *P* < 0.001; δ^15^N: *H* = 25.203, df = 3, *P* < 0.001) (Fig. 2a; pairwise comparisons Table S1). The dual isotope approach supports an isotopic separation of hyphae from soil in ^15^N and hyphae from plant in ^13^C and ^15^N.

Higher transpiration rates and lower water use efficiency in the C_3_ monocot grass relative to the dicotyledon legume (Rawson et al. 1977; Adams et al. 2016) likely resulted in significantly δ^13^C enriched legume leaves relative to grass leaves (*U*(12,12) = 135, *P* < 0.001), while more depleted legume leaf δ^15^N values (*U*(12,12) = 32, *P* = 0.020) relative to the grass may indicate a contribution of the N-fixing bacteria within the 78-day growth period, although the soil was sterilized at the beginning. Mycorrhizal fungi transfer mineral nutrients via their hyphae to their plant partners in exchange for carbohydrates originating from photosynthesis (Wipf et al. 2019). The ^13^C enrichment of AM hyphae potentially results from the gain of ^13^C-enriched carbohydrates from the plant (cf. Gleixner et al. 1993). Additionally, AM fungi lack the ability of lipid synthesis. Thus, lipids originated from the plant partner (Luginbuehl et al. 2017; Jiang et al. 2017; Keymer et al. 2017; Rich et al. 2017) likely represent a supplemental carbon source. Lipids were shown to be ^13^C-depleted relative to bulk tissue by Gleixner et al. (1993). We here detected ^13^C enrichment of AM soil hyphae and intraradical hyphae of about 6‰ relative to the plant which is consistent with findings on AM soil hyphae by Walder et al. (2012), but also is less pronounced than the relative ^13^C enrichment of ectomycorrhizal fruiting bodies relative to their associated plants of about 6–10‰ (cf. Trudell et al. 2004; Gebauer et al. 2016; Schiebold et al. 2017; Chen et al. 2019). While ectomycorrhizal fungi mostly rely on plant-derived carbohydrates, the ^13^C enrichment of AM fungi appears to be counterbalanced by a mixture of two carbon sources, plant-originated carbohydrates and lipids. Walder et al. (2012) claimed contamination by soil particles could not be excluded while the ^13^C and ^15^N dual isotope approach utilized here supports an isotopic separation of hyphae relative to soil.

Arbuscular mycorrhizal fungi are assumed to possess limited saprotrophic capability (Nakano et al. 1999; Smith and Read 2008; Tisserant et al. 2013) which should ultimately result in the utilization of similar inorganic nitrogen sources as the plant partner. Therefore, an insignificant difference in ^15^N natural abundance between plant and AM hyphae was expected. To the contrary, our data show an ^15^N enrichment for the AM hyphae relative to plant leaf material by *c.* 4.2‰. Perhaps, the AM fungi also might utilize low molecular weight organic nitrogen sources that possibly were released from bacterial biomass by the steam-sterilizing process. A labile organic nitrogen source might explain the ^15^N enrichment, despite translocation of ammonium and nitrate to the plants. This is concurrent with earlier analysis by Gebauer and Dietrich (1993) on ectomycorrhizas. Nonetheless, a part of the ^15^N enrichment is likely still a result of trophic enrichment (DeNiro and Epstein 1981, Peterson and Fry 1987). The gain of nitrogen from organic material (cf. Hodge et al. 2001; Leigh et al. 2009) might be dependent on AMF species and strain.

Furthermore, our findings of ^13^C and ^15^N enrichment support studies of mycoheterotrophic plants and their AM fungi, which so far may have been compromised by surrogates for AM fungal isotopic signatures. Mycoheterotrophic plants (MHP) partially or completely cover their carbon demand from a fungal source (Hynson et al. 2013; Merckx 2013). The MHP leaves’ ^15^N patterns were found to be determined by the MHP’s root fungi (Schiebold et al. 2017). Hitherto, ^13^C and frequently ^15^N enrichments of MHP with AM fungi were associated with fungal identity, different fungal communities and different geographic origin (Merckx et al. 2010; Courty et al. 2011; Giesemann et al. 2020; Gomes et al. 2020). The previous lack of proof of ^13^C and ^15^N enrichment of the AM fungus itself was a major point of limitation, which can now be addressed with the methods described here.

### Practicability of AM hyphal isotopic patterns

Several studies have shown successful accessing of AM fungal spores and specific fungal compounds, such as PLFA/NLFA 16:1ω5, for stable isotope analyses (Allen and Allen 1990; Nakano et al. 1999; Courty et al. 2011; Walder et al. 2012, 2013; Courty et al. 2015; Suetsugu et al. 2020). Nevertheless, spores and PLFAs/NLFAs biomarkers often show different isotopic signatures than hyphae. Spores’ range from a 1.5 to 5.2‰ ^13^C depletion relative to the root (Allen and Allen 1990; Nakano et al. 1999; Walder et al. 2012) and 1.2 to 4.9‰ ^13^C enrichment relative to the plant (Courty et al. 2011; Suetsugu et al. 2020). PLFA C16:1ω5 was approx. 2.4‰ more ^13^C-depleted than roots (Walder et al. 2013; Ven et al. 2020) and NLFA C16:1ω5 was approx. 3.7‰ more ^13^C-depleted than roots (Ven et al. 2020). In contrast, AM hyphae have previously been shown to be continuously ^13^C-enriched by around 5‰ (Walder et al. 2012, and this study) relative to plant leaves, a pattern also found for other mycorrhizas (ECM) (e.g. Gebauer et al. 2016; Chen et al. 2019). Thus, when isotopic data on hyphae as the active nutrient pathway are required in future research, the approach presented here could be considered.

Isolation of coenocytic AM hyphae bears the risk of loss of cytoplasm and the inclusion of bacterial biofilms. In our study, the aim was to minimize the risk of isotopic fractionation induced by chemicals, wherefore the vitality of hyphae was neglected. Applying the approach presented in Saito (1995) allows for the gain of living hyphae (due to the presence of several buffers and solutions) with minimized loss of cytoplasm. Eventually, the trade-off between intact, living hyphae or the minimization of chemical-induced isotopic fractionation needs to be evaluated. An impact of bacterial biofilms adhering to hyphal surfaces cannot be excluded completely. Nevertheless, this impact should be present, despite varying bacterial diversity, for soil hyphae and plant roots (Marilley et al. 1998; Roesch et al. 2008), plus potentially for AM fungal spores. In addition, an influence of bacteria might also occur in PLFA analyses, as the frequently applied AM fungi–specific PLFA C16:1ω5 was also found in some Gram-negative bacteria and can be affected by degrading spores (Nichols et al. 1986; Joergensen and Wichern 2008; Ngosong et al. 2012; Paterson et al. 2016). NLFA 16:1ω5 are considered more specific to AM fungi than PLFA 16:1ω5 as they accumulate as a carbon storage compound in biomass (Olsson et al. 2005) and the ratio between NLFA and PLFA 16:1ω5 is higher in AM fungi than in bacteria (Olsson 1999). However, background NLFA 16:1ω5 concentrations of spores and free neutral lipids from non-living biomass with a long residence time also were described for these biomarkers (Paterson et al. 2016). Lately, the absence of a completely AM fungi–specific fatty acid and differences in the fatty acid composition between AM fungal species and genera need to be considered (Olsson 1999). While both AM hyphae and biomarkers can be affected by bacteria, PLFA/NLFA biomarkers for ^13^C AM isotopic data may be supplemented by ^15^N isotope natural abundance signatures of the AM hyphae.

When aiming to transfer the approaches presented here for isolation of AM fungal hyphae to field trials, the question of separation from other fungal groups occurs. For soil hyphae, AMF could be separated from other fungal groups under the dissecting microscope via the presence or absence of septa (Smith and Read 2008). Choosing the isolation of intraradical hyphae from roots potentially reduces the diversity of other fungal groups than AM fungi relative to soil (Gao et al. 2019; Mahmoudi et al. 2019) due to plants’ selection mechanisms, while potentially increasing the AM fungal diversity (Mahmoudi et al. 2019). To increase chances of high AM fungal colonization, multiple fine-root fragments instead of single long roots should be sampled (cf. Mahmoudi et al. 2019).

### Outlook

The constancy of stable isotope ^13^C and ^15^N patterns needs to be analysed on a broader scale and along different genera, species and strains of AM fungi. Assuming the existence of relatively defined isotopic variability within AM fungi, the comparison of dual or multi-isotope patterns of different fungal groups may represent a possibility to estimate the contribution of fungal groups to nutrient trading within mycorrhizal networks, especially in field trials. Picking-up the C_3_-C_4_-Common Mycorrhizal Network approach conducted by Walder et al. (2012), (2013) and Courty et al. (2015), the analysis of intraradical hyphal isotopic patterns complementary to soil hyphae, spores or biomarkers might reveal an interesting perspective. Finally, the possibility of intraradical hyphae extraction might further support research on AM mycoheterotrophic plants and shed light on the insufficiently known function of difficult to access ascomycotan dark septate endophytes or basidiomycotan *Rhizoctonia*-like fungi.

## Electronic supplementary material

ESM 1 (DOCX 36.4 kb)

## Data Availability

The single δ^13^C, δ^15^N, N-content and C:N ratio values can be obtained from the supplemental material (Table S3).
