# Supplementary material for: Stable C and N isotope natural abundances of intraradical hyphae of arbuscular mycorrhizal fungi
Source: Mycorrhiza. 2020 Aug 25;30(6):773–80. doi: 10.1007/s00572-020-00981-9 (PMC7591432; doi:10.1007/s00572-020-00981-9)
Supplement: Supplementary file 1 — (DOCX 36.4 kb) [file 572_2020_981_MOESM1_ESM.docx]

**Table S1** All pairwise multiple comparison (Pairwise Wilcoxon *post hoc* test with ‘Holm-Bonferroni correction’) of hyphae to leaves, roots and soil from either *Festuca ovina* or *Medicago sativa* rhizoboxes in δ^13^C and δ^15^N. Significant differences are highlighted in bold. Note, since soil hyphae and root hyphae showed similar δ^13^C and δ^15^N values we combined these for comparison with plant tissues and soil

|  | **Sample (*N*)** | δ^13^C | | δ^15^N | |
| --- | --- | --- | --- | --- | --- |
|  |  | ***Z*** | ***P*** | ***Z*** | ***P*** |
| *Medicago*  *rhizoboxes* | Leaves (*12*) *vs*. hyphae (6) | - 3.3335 | **0.0051** | - 3.3266 | **0.0053** |
|  | Roots (*6*) *vs*. hyphae (6) | - 2.8022 | **0.0152** | - 2.8022 | **0.0203** |
|  | Soil (*6*) *vs*. hyphae (6) | - 0.8807 | 0.3785 | -2.8022 | **0.0203** |
|  | Leaves (*12*) *vs*. roots (*6*) | - 3.3249 | **0.0053** | - 1.0313 | 0.3024 |
|  | Leaves (*12*) *vs*. soil (*6*) | - 3.3249 | **0.0053** | - 3.3266 | **0.0044** |
|  | Roots (*6*) *vs*. soil (*6*) | - 2.8022 | **0.0175** | - 2.8022 | **0.0101** |
| *Festuca rhizoboxes* | Leaves (*12*) *vs*. hyphae (6) | - 3.3283 | **0.0052** | - 3.3283 | **0.0052** |
|  | Roots (*6*) *vs*. hyphae (6) | - 2.8022 | **0.0148** | - 2.8022 | **0.0203** |
|  | Soil (*6*) *vs*. hyphae (6) | - 2.8121 | **0.0148** | - 2.8022 | **0.0203** |
|  | Leaves (*12*) *vs*. roots (*6*) | - 3.3249 | **0.0053** | - 2.7658 | **0.0101** |
|  | Leaves (*12*) *vs*. soil (*6*) | - 3.3249 | **0.0053** | - 3.3283 | **0.0044** |
|  | Roots (6) *vs*. soil (6) | - 2.8022 | **0.0175** | - 2.8022 | **0.0101** |

**Table S2** Mann-Whitney *U* tests of *Agaricus* *bisporus* fruiting bodies treated with enzyme solution or sodium-hexametaphosphate relative to a non-treated *Agaricus* control. The effect of the chemicals used was statistically evaluated in ^13^C and ^15^N. Note that different sporocarps of *Agaricus bisporus* were used (I-IV)

|  |  | δ^13^C | | δ^15^N | |
| --- | --- | --- | --- | --- | --- |
|  |  | ***U*** | ***P*** | ***U*** | ***P*** |
|  | **Fruiting Bodies (*N*)** |  |  |  |  |
|  | Control I (*3*) *vs*. enzyme I (*3*) | 8 | 0.190 | 9 | 0.081 |
|  | Control II (*3*) *vs*. enzyme II (*3*) | 2 | 0.383 | 6 | 0.663 |
|  | Control III (*3*) *vs*. enzyme III (*3*) | 4 | 1.000 | 9 | 0.081 |
|  | Control IV (*3*) *vs*. SHMP IV (*3*) | 7 | 0.860 | 10 | 0.216 |

SHMP: sodium-hexametaphosphate

**Table S3** Single δ^13^C values, δ^15^N values, N-content [mmol g dwt^-1^] and C:N ratio of AM hyphae, plant leaves, roots, soil and fruiting bodies of *Agaricus bisporus*. Hyphae measured at µEA-IRMS are indicated by an asterix (*). Note that different sporocarps were used for *Agaricus bisporus*, but always the same for a control and treatment comparison

| **Tissue/compartment** | **δ^13^C [‰]** | **δ^15^N [‰]** | **N-content  [mmol g dwt^-1^]** | **C:N ratio** |
| --- | --- | --- | --- | --- |
| *Festuca* soil hyphae* | -27.31 | 6.77 | 2.17 | 11.72 |
| *Festuca* soil hyphae* | -27.48 | 5.19 | 3.14 | 10.80 |
| *Festuca* hyphae. mechanical* | -27.01 | 5.67 | 1.85 | 10.78 |
| *Festuca* hyphae. mechanical* | -27.18 | 4.91 | 4.92 | 10.24 |
| *Medicago* soil hyphae* | -28.04 | 5.37 | 1.41 | 14.61 |
| *Medicago* soil hyphae* | -27.20 | 5.01 | 4.80 | 10.36 |
| *Medicago* hyphae. mechanical* | -27.45 | 5.80 | 3.98 | 10.56 |
| *Medicago* hyphae. mechanical* | -27.49 | 5.27 | 2.58 | 10.18 |
| *Festuca* soil hyphae | -27.21 | NA | NA | NA |
| *Festuca* soil hyphae | -27.75 | NA | NA | NA |
| *Festuca* soil hyphae | -27.07 | NA | NA | NA |
| *Festuca* soil hyphae | -27.26 | 5.63 | 2.19 | 13.49 |
| *Festuca* hyphae. mechanical | -27.13 | NA | NA | NA |
| *Festuca* hyphae. mechanical | -27.13 | 6.73 | 2.03 | 9.87 |
| *Festuca* hyphae. mechanical | -27.63 | NA | NA | NA |
| *Festuca* hyphae. mechanical | -26.95 | NA | NA | NA |
| *Festuca* hyphae. enzymatic | -27.71 | NA | NA | NA |
| *Festuca* hyphae. enzymatic | -27.26 | NA | NA | NA |
| *Festuca* hyphae. enzymatic | -26.19 | NA | NA | NA |
| *Festuca* hyphae. enzymatic | -26.21 | NA | NA | NA |
| *Medicago* soil hyphae | -27.12 | NA | NA | NA |
| *Medicago* soil hyphae | -26.47 | NA | NA | NA |
| *Medicago* soil hyphae | -27.84 | 5.41 | 2.14 | 13.01 |
| *Medicago* soil hyphae | -27.27 | NA | NA | NA |
| *Medicago* hyphae. mechanical | -27.18 | NA | NA | NA |
| *Medicago* hyphae. mechanical | -27.13 | 5.67 | 2.30 | 9.92 |
| *Medicago* hyphae. mechanical | -27.75 | NA | NA | NA |
| *Medicago* hyphae. mechanical | -27.96 | NA | NA | NA |
| *Medicago* hyphae. enzymatic | -27.99 | NA | NA | NA |
| *Medicago* hyphae. enzymatic | -26.28 | NA | NA | NA |
| *Medicago* hyphae. enzymatic | -28.02 | NA | NA | NA |
| *Medicago* hyphae. enzymatic | -26.75 | NA | NA | NA |
| *Festuca* leaves | -33.28 | 0.24 | 1.67 | 17.11 |
| *Festuca* leaves | -33.72 | 0.49 | 1.63 | 18.84 |
| *Festuca* leaves | -34.50 | 1.68 | 1.84 | 17.77 |
| *Festuca* leaves | -34.62 | 1.65 | 1.87 | 17.67 |
| *Festuca* leaves | -34.58 | 1.56 | 1.87 | 17.49 |
| *Festuca* leaves | -34.62 | 1.71 | 1.87 | 17.75 |
| *Festuca* leaves | -34.69 | 1.81 | 1.90 | 17.52 |
| *Festuca* leaves | -34.42 | 1.71 | 1.81 | 18.44 |
| *Festuca* leaves | -34.10 | 1.43 | 1.74 | 19.01 |
| *Festuca* leaves | -34.16 | 1.50 | 1.78 | 18.63 |
| *Festuca* leaves | -34.50 | 1.55 | 1.86 | 17.80 |
| *Festuca* leaves | -34.66 | 1.65 | 1.90 | 17.50 |
| *Medicago* leaves | -33.27 | 0.94 | 1.85 | 18.35 |
| *Medicago* leaves | -33.27 | 0.93 | 1.75 | 18.36 |
| *Medicago* leaves | -33.25 | 0.65 | 1.78 | 18.69 |
| *Medicago* leaves | -33.74 | 1.25 | 3.44 | 9.77 |
| *Medicago* leaves | -33.70 | 1.73 | 3.45 | 9.75 |
| *Medicago* leaves | -33.70 | 1.24 | 3.44 | 9.77 |
| *Medicago* leaves | -33.71 | 1.13 | 3.46 | 9.70 |
| *Medicago* leaves | -33.52 | 0.93 | 3.65 | 9.36 |
| *Medicago* leaves | -33.64 | 0.86 | 3.66 | 9.32 |
| *Medicago* leaves | -33.59 | 0.77 | 3.64 | 9.36 |
| *Medicago* leaves | -33.69 | 1.04 | 3.67 | 9.31 |
| *Medicago* leaves | -33.27 | 0.24 | 3.21 | 9.81 |
| *Festuca* roots | -32.31 | 1.18 | 0.89 | 27.97 |
| *Festuca* roots | -32.42 | -0.04 | 1.60 | 17.51 |
| *Festuca* roots | -32.55 | 0.88 | 1.59 | 18.63 |
| *Festuca* roots | -32.50 | 0.96 | 2.08 | 14.62 |
| *Festuca* roots | -31.63 | -0.68 | 1.43 | 18.94 |
| *Festuca* roots | -31.64 | 0.13 | 2.02 | 15.38 |
| *Medicago* roots | -31.79 | 0.86 | 2.23 | 14.18 |
| *Medicago* roots | -32.08 | 1.14 | 1.85 | 15.47 |
| *Medicago* roots | -31.96 | 0.19 | 1.64 | 16.68 |
| *Medicago* roots | -32.16 | 1.30 | 1.87 | 15.08 |
| *Medicago* roots | -31.69 | 0.51 | 2.07 | 15.02 |
| *Medicago* roots | -31.53 | 0.03 | 1.87 | 15.21 |
| *Festuca* soil | -27.71 | 2.65 | 0.05 | 13.55 |
| *Festuca* soil | -27.79 | 3.46 | 0.05 | 12.63 |
| *Festuca* soil | -27.58 | 2.85 | 0.05 | 13.09 |
| *Festuca* soil | -27.71 | 2.97 | 0.05 | 13.64 |
| *Festuca* soil | -27.58 | 3.15 | 0.05 | 13.03 |
| *Festuca* soil | -27.65 | 2.86 | 0.05 | 13.30 |
| *Medicago* soil | -27.73 | 3.42 | 0.05 | 13.36 |
| *Medicago* soil | -27.61 | 3.28 | 0.05 | 13.56 |
| *Medicago* soil | -27.68 | 3.18 | 0.05 | 13.36 |
| *Medicago* soil | -27.70 | 3.43 | 0.05 | 13.14 |
| *Medicago* soil | -27.54 | 2.74 | 0.04 | 14.14 |
| *Medicago* soil | -27.56 | 2.84 | 0.05 | 13.80 |
| *Agaricus* control I | -23.90 | 17.10 | 2.89 | 9.86 |
| *Agaricus* control I | -23.44 | 17.90 | 3.03 | 9.26 |
| *Agaricus* control I | -23.17 | 17.87 | 3.35 | 8.60 |
| *Agaricus* enzyme I | -24.19 | 14.84 | 3.73 | 8.40 |
| *Agaricus* enzyme I | -23.63 | 16.31 | 4.05 | 7.91 |
| *Agaricus* enzyme I | -24.42 | 15.85 | 4.10 | 7.86 |
| *Agaricus* control II | -25.33 | 5.04 | 8.26 | 10.44 |
| *Agaricus* control II | -23.16 | 12.34 | 4.05 | 7.07 |
| *Agaricus* control II | -23.19 | 11.72 | 4.21 | 6.89 |
| *Agaricus* enzyme II | -23.10 | 10.03 | 3.45 | 7.88 |
| *Agaricus* enzyme II | -23.62 | 10.04 | 3.46 | 9.36 |
| *Agaricus* enzyme II | -23.08 | 9.78 | 3.64 | 7.61 |
| *Agaricus* control III | -22.63 | 11.58 | 3.60 | 7.56 |
| *Agaricus* control III | -22.42 | 21.83 | 4.20 | 6.65 |
| *Agaricus* control III | -22.71 | 11.96 | 3.77 | 7.10 |
| *Agaricus* enzyme III | -22.52 | 9.30 | 3.19 | 8.00 |
| *Agaricus* enzyme III | -22.78 | 9.41 | 3.09 | 8.13 |
| *Agaricus* enzyme III | -22.32 | 9.34 | 3.67 | 7.40 |
| *Agaricus* control IV | -21.70 | 19.52 | 6.82 | 4.42 |
| *Agaricus* control IV | -22.02 | 18.45 | 5.90 | 4.95 |
| *Agaricus* control IV | -22.62 | 17.59 | 5.37 | 5.48 |
| *Agaricus* Sodium-Hexametaphosphate | -22.57 | 11.81 | 4.12 | 6.67 |
| *Agaricus* Sodium-Hexametaphosphate | -22.61 | 17.57 | 5.25 | 5.67 |
| *Agaricus* Sodium-Hexametaphosphate | -22.47 | 17.33 | 5.17 | 5.79 |
| *Agaricus* Sodium-Hexametaphosphate | -21.83 | 18.51 | 5.59 | 5.52 |
